# Outcomes of intra-articular calcaneal fractures: surgical treatment of 114 consecutive cases at a maximum care trauma center

**DOI:** 10.1186/s12891-021-04088-w

**Published:** 2021-03-01

**Authors:** Christin Schindler, Andreas Schirm, Vilijam Zdravkovic, Primoz Potocnik, Bernhard Jost, Andreas Toepfer

**Affiliations:** 1 Department of Orthopaedics and Traumatology, Kantonsspital St. Gallen, Rorschacher Strasse 95, 9007 St. Gallen, Switzerland; 2 Hand und Fuss Facharztpraxis, Pestalozzistr.2, Zentrum St. Leonhard, 9000 St. Gallen, Switzerland

**Keywords:** Calcaneal fracture, Operative treatment, Outcome, Subtalar fusion

## Abstract

**Background:**

The aim of this retrospective monocentric study was to investigate the outcomes of surgically treated intra-articular calcaneus fractures in a maximum care trauma center.

**Methods:**

One hundred forty patients who had undergone surgery for intra-articular calcaneal fractures between 2002 and 2013 were included. One hundred fourteen cases with 129 fractures were eligible to participate in the study of which 80 were available for a clinical and radiological follow-up. 34 patients were followed up by telephone interview only. Outcome measures included the American Orthopaedic Foot and Ankle Society (AOFAS) hindfoot score, Short Form 36 Health Status Survey (SF-36), complications, and subsequent surgeries.

**Results:**

Mean follow-up was 91 months (range 12–183). The overall complication rate was 29% (37/129 ft). Disturbed wound healing (11%) and infection (5%) occurred most commonly. Non-union (4%) only occurred in smokers (*p* = 0.02). A high rate of posttraumatic subtalar arthritis (77%) and need for subsequent subtalar fusion (18%) without independent risk factors for subsequent subtalar fusion was found. The revision rate was high (60%) after primary fusion. Mean AOFAS-hindfoot score was 74 (Sanders I: 99, Sanders II: 74, Sanders III: 77, Sanders IV: 70). The postoperative Boehler angle improved significantly in all subgroups (*p* < 0.01). Patients with a decreased Boehler angle between postoperative images and the follow-up had significantly lower AOFAS hindfoot scores (*p* < 0.01).

**Conclusions:**

Our data can aid decision-making in the treatment of calcaneal fractures. We advocate to use primary subtalar fusion with caution due to the high revision rate. Smoking status should always be considered.

Level of evidence: Level III, retrospective cohort study.

## Background

The calcaneus is the most commonly fractured tarsal bone accounting for 1–2% of all fractures [1, 2]. Seventy-five percent of all calcaneal fractures involve the subtalar joint [3]. The injury is most often caused by falling from a height [4, 5]. Young male workers are frequently affected, which explains the high socio-economic burden of this injury [1, 5]. Intra-articular calcaneal fractures are known to have an unfavourable outcome [6]. The impact of this injury on personal well-being has been shown to be higher than that of myocardial infarction [6]. The optimal treatment has been subject to debate for many decades and until today no commonly accepted consensus found [7]. Even the existing randomized controlled trials on operative vs. nonoperative treatment of the last 25 years failed to establish clarity. Parmar et al. 1993, Ibrahim et al. 2007 and Sharma et al. 2011 found no significant differences between their operative and nonoperative groups [8–10]. Thordarson and Krieger 1996, Rodriguez-Merchan et al. 1999, Howard et al. 2003 and Bahari et al. 2013 had better functional results in their operative group [11–14]. Some studies did show better outcomes in a subgroup of their operated patients: Buckley et al. 2002 in the group without workers´ compensation; Nouraei and Moosa 2011 in operatively treated patients without open fractures, osteoporosis, poor general health, or severe comminution [15, 16]. Griffin et al., on the other hand, found higher complication rates in the operative group and did not recommend operative treatment at all [2]. Agren et al. showed a higher complication rate as well but a lower incidence of posttraumatic arthritis [17].

Sanders et al. found a correlation between the need for subsequent subtalar arthrodesis and Sanders classification in their long-term study, where patients with a Sanders III fracture were 4 times more likely to need a subtalar fusion than patients with a Sanders II fracture [18]. The long-term study by Rammelt et al. also showed worse functional outcomes in patients with higher fracture severity [19]. This study was conducted to investigate the relationship between fracture severity, method of operative treatment, and outcome. We were searching for independent risk factors for the need for subtalar arthrodesis.

## Methods

As part of a monocentric retrospective study, we identified all patients who had sustained intra-articular calcaneal fractures between February 2002 and August 2013 and were treated operatively. Criteria for surgical fixation were: posterior facet step-off of more than 2–3 mm, flattening of Bohlers angle, coronal plane malalignment of the tuberosity, and large laterally displaced fragments that would lead to subfibular impingement. Exclusion criteria were extra-articular fractures, conservative treatment, and known pre-existing osteoarthritis of the subtalar joint. Ethics committee approval and informed consent were obtained. All eligible patients were invited to be examined clinically and radiologically. This involved gait assessment, hindfoot alignment, tenderness to palpation, swelling, heel width, and subtalar range of motion (ROM).

Patient history was documented including: injury mechanism, age at the time of injury, gender, injured side, work-related accident, occupation, return to work, concomitant injuries, delay until surgery, surgical technique, subsequent operations, complications, symptoms of arthritic changes (weather-dependent discomfort, morning stiffness, night pain), swelling, pre-existing conditions (smoking, diabetes, osteoporosis, steroids), use of medical aids (insoles, orthopedic shoes) and difference in shoe size. The American Orthopaedic Foot and Ankle Society (AOFAS) hindfoot score was completed and all patients were asked to answer the Short Form 36 Health Status Survey (SF-36). Quality Metric Health Outcomes scoring software 5.0 was used to collect, evaluate, and score SF-36 data. X-rays were taken of the feet involved and assessed for signs of subtalar osteoarthritis. Fracture severity was classified according to Sanders with the initial CT scan. Boehler angles were measured at the time of injury, postoperatively, and at the latest follow-up. Patients unable or unwilling to attend the clinical and radiological examination were asked to participate in a telephone interview.

All statistical analyses were performed with *R* (R Foundation for Statistical Computing, Vienna, Austria. URL http://www.R-project.org/). Descriptive statistics included means, standard deviations, ranges, and proportions. Comparative statistics included t-tests and the Chi-square test (where appropriate the Fisher exact test was applied). The level of significance was set at *p* < 0.05.

## Results

**Fig. 1:**
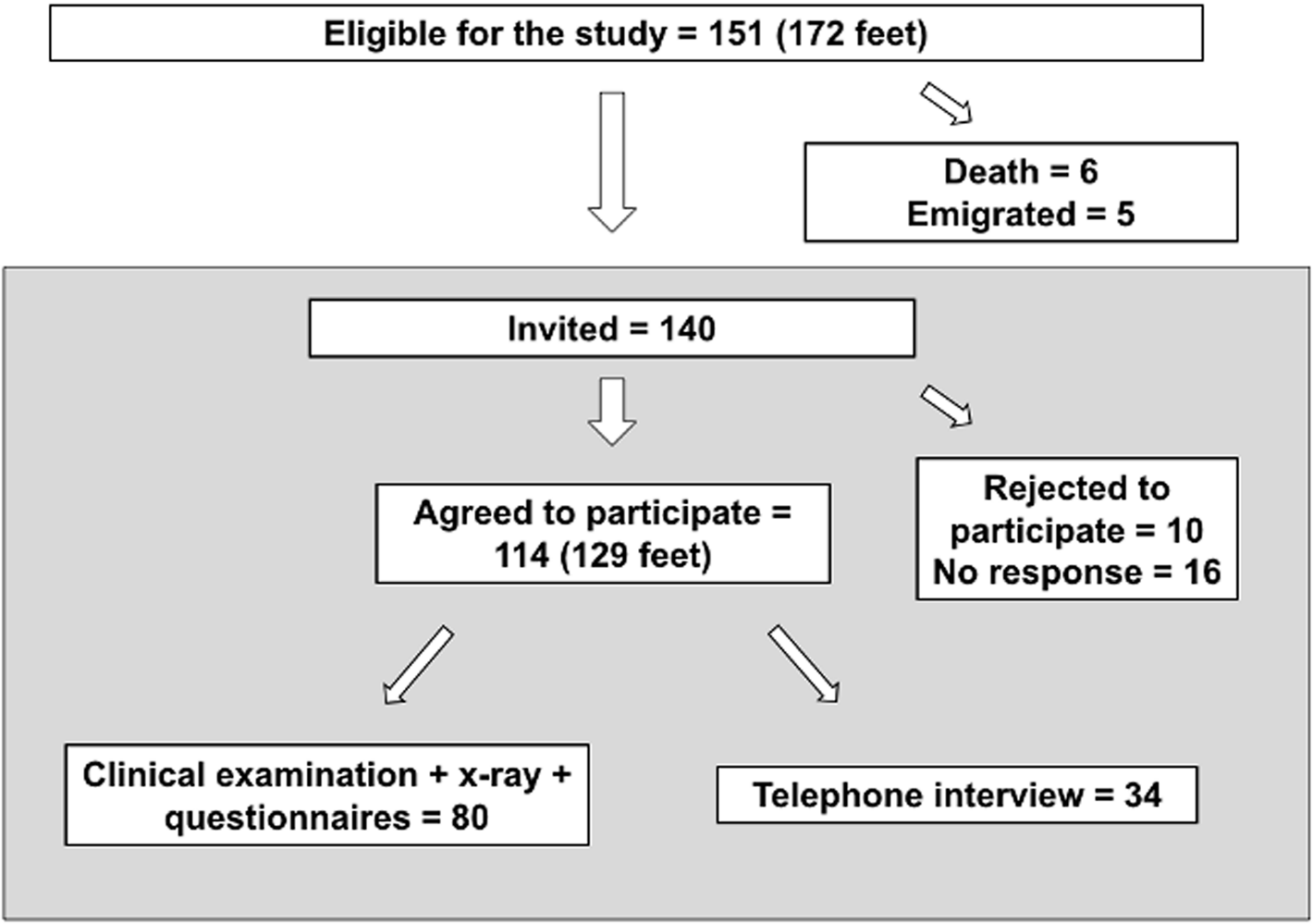
Patient selection flow chart

Figure 1 shows patient flow: 114 patients with 129 operated feet (62 right, 67 left feet). The mean follow-up was 91 months (range 12–183 months). Table 1 shows patient demographics.

**Table 1:** Patient demographics

	all	male	female
Patients	114	83	31
Mean age (range)	41.5 (11–83)	41.2 (11–75)	45.6 (15–83)
Cause of injury			
Fall from height	90	67	23
Sport injury	17	15	2
MVA	15	10	5
other	7	3	4
Work-related	34	29	5
Not work-related	80	54	26

MVA: motor vehicle accident.

Fracture severity according to the Sanders classification was distributed as shown in Table 2.

**Table 2:** Sanders classification

Sanders	I	II			III			IV
		IIA	IIB	IIC	IIIAB	IIIAC	IIIBC	
		6	11	1	18	9	5	
total	3	18			32			52

The three Sanders Type I fractures that were treated operatively were tongue type fractures with an undisplaced intraarticular extension. Twenty-four fractures could not be classified retrospectively because the preoperative CT scans were not available. Four fractures were open (2 Sanders IV, 1 IIIBC, 1 unknown). Two of the open fractures occurred in patients with bilateral calcaneal fractures. Fifty-one patients sustained concomitant injuries (Table 3).

**Table 3:** Concomitant injuries

Concomitant Injuries	Sanders I	Sanders II	Sanders III	Sanders IV	Not classified	Total
None	0	12	23	19	13	67
Spine	1	1	4	11	3	20
Ipsilateral foot/lower extremity	1	3	1	8	5	18
Contralateral calcaneus operative	0	0	2	11	2	15
Contralateral calcaneus nonoperative	0	2	2	4	1	9
Others	1	1	6	15	4	27
Multiple concomitant injuries	0	1	3	12	3	19

Patients with Sanders IV fractures suffered from concomitant injuries significantly more often (*p* < 0.01). The AOFAS hindfoot score was significantly worse (*p* = 0.02) in the presence of concomitant injuries.

Preexisting medical conditions were uncommon among our patients. Two patients had diabetes, two were on oral steroids and two had previously diagnosed osteoporosis. Steroid medication was used due to rheumatoid arthritis in one and chronic obstructive lung disease in the other patient. Forty-seven patients (41%) stated that they were smokers at the time of the accident and perioperatively. The detailed number of pack-years (py) was only obtainable in 26 patients (mean 22 py, range 4–40 py).

**Fig. 2:**
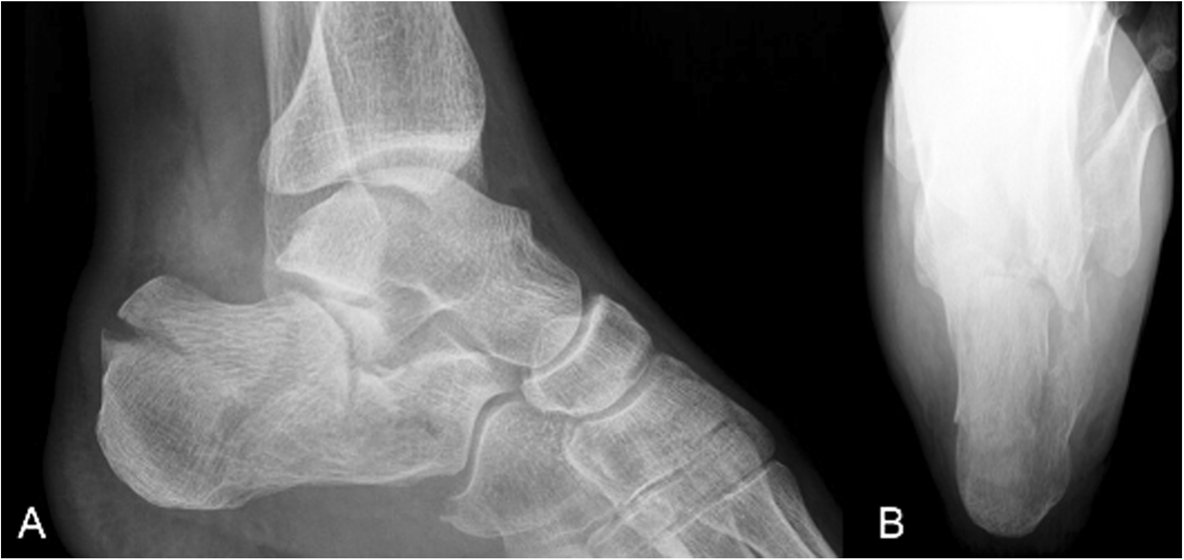
Case 1 Preoperative radiographs. Radiographs left calcaneus lateral (A) and axial (B) view, 62 year old male patient, non-smoker

**Fig. 3:**
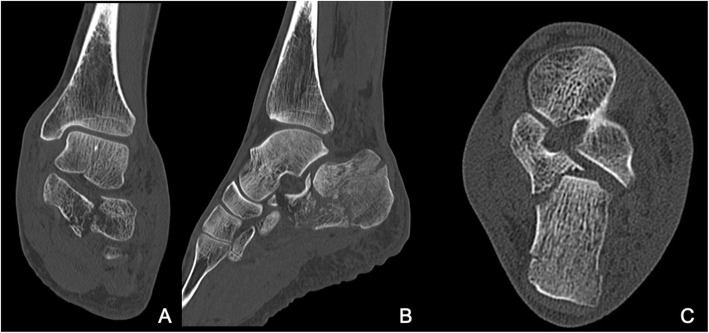
Case 1 Preoperative CT. Coronal (A), sagittal (B) and axial (C) views representing a Sanders IIb fracture type pattern

**Fig. 4:**
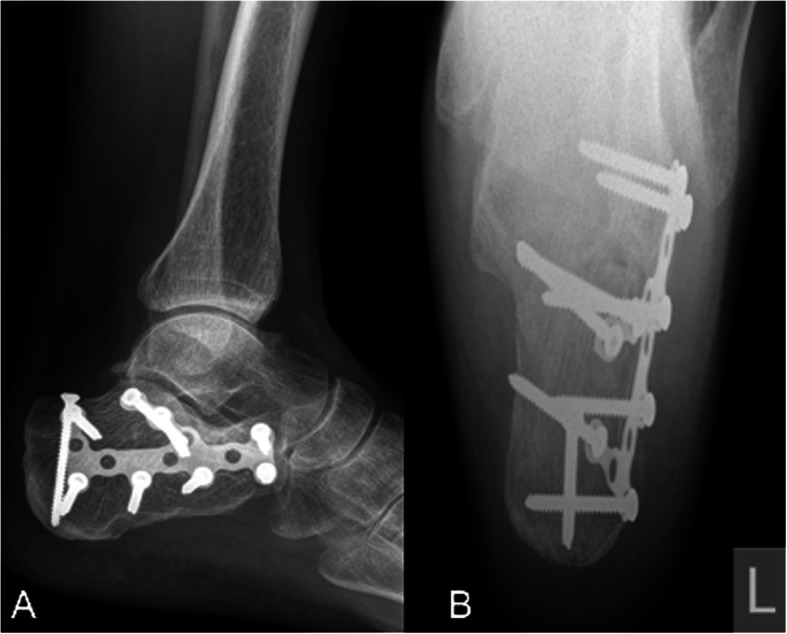
Case 1 Postoperative radiographs. Lateral (A) and axial (B) radiographs after ORIF via an extended lateral approach

**Fig. 5:**
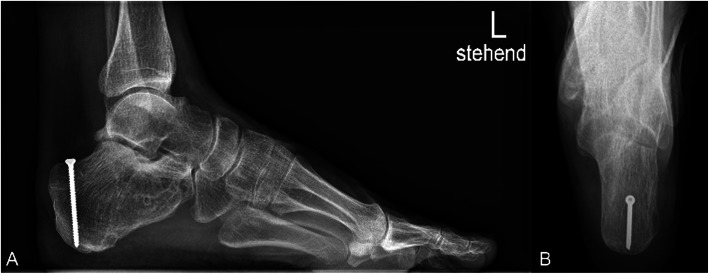
Case 1 Radiographs at follow-up Weightbearing left foot lateral (A) and calcaneus axial (B) 40 months post-surgery, AOFAS score 95

The mean time between the accident and the definitive surgery was 8.3 days (range 0–38 days). Reasons for delay in surgery included waiting for subsidence of swelling and fracture blisters, delayed surgery of non-life-threatening injuries in polytrauma patients, definite surgery after repatriation from abroad. One hundred two fractures were treated with open reduction and internal plate fixation (ORIF) through an extended lateral approach. The implant used for osteosynthesis was either the Synthes Locking Calcaneal Plate (DePuy Synthes Medical Devices, Oberdorf, Switzerland) or the Mondeal Calcaneus Plate (Mondeal Medical Systems GmbH, Muehlheim, Germany). Other methods (K-wires, Ex-fix, screw fixation) were used in 22 fractures. One patient with bilateral Sanders IV fractures developed compartment syndrome in both feet prior to surgical fixation and was treated with foot compartment release. In total, ten different board-certified trauma surgeons performed the surgeries over a period of 11 years. Figures 2, 3, 4 and 5 show a case of a Sanders II fracture treated with ORIF.

**Fig. 6:**
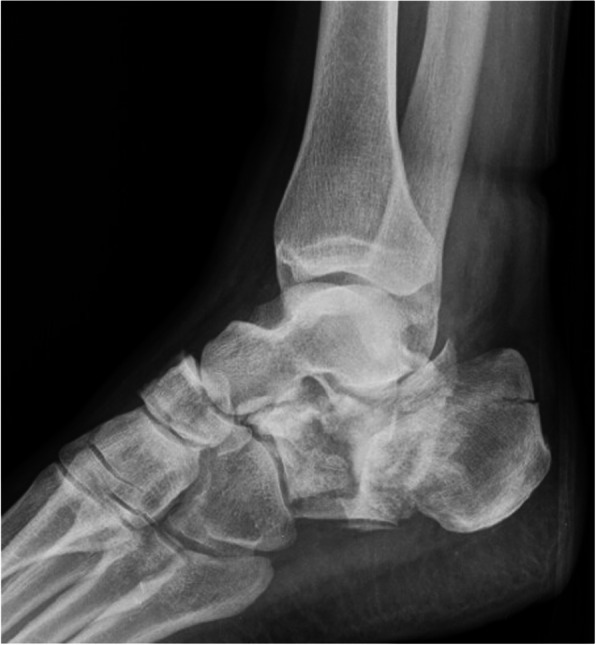
Case 2 Preoperative radiographs. Radiograph right calcaneus lateral, 35 year old female patient, smoker

**Fig. 7:**
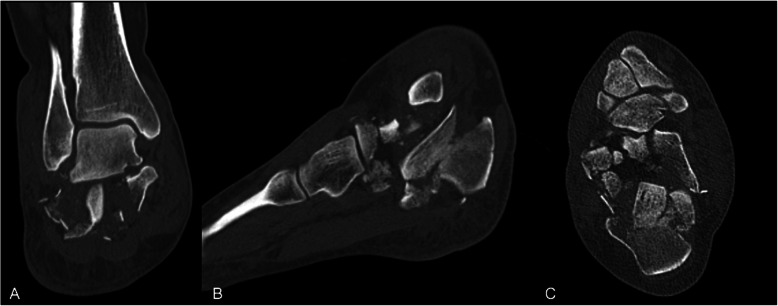
Case 2 Preoperative CT Coronal (A) sagittal (B) and axial (C) views representing a Sanders IV fracture type pattern

**Fig. 8:**
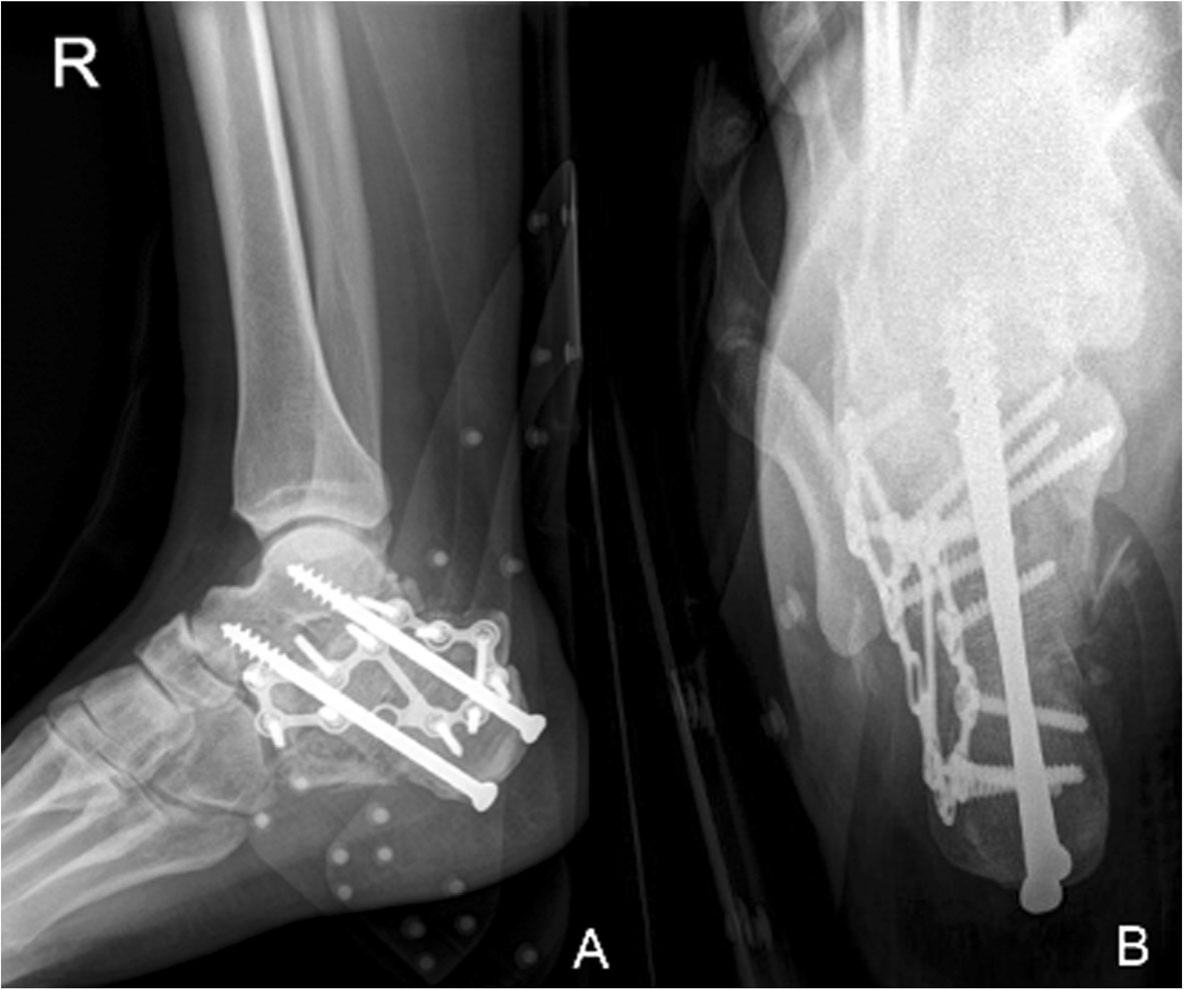
Case 2 Postoperative radiographs Lateral (A) and axial (B) radiographs after primary subtalar arthrodesis

**Fig. 9:**
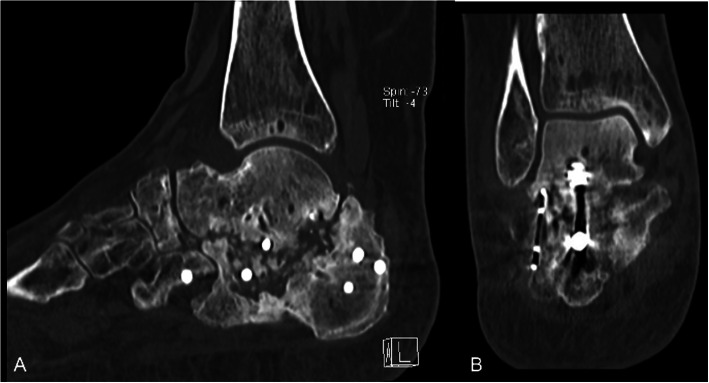
Case 2 Complications. CT scan sagittal (A) and coronal (B) view 6 months after primary subtalar fusion showing a subtalar non-union

**Fig. 10:**
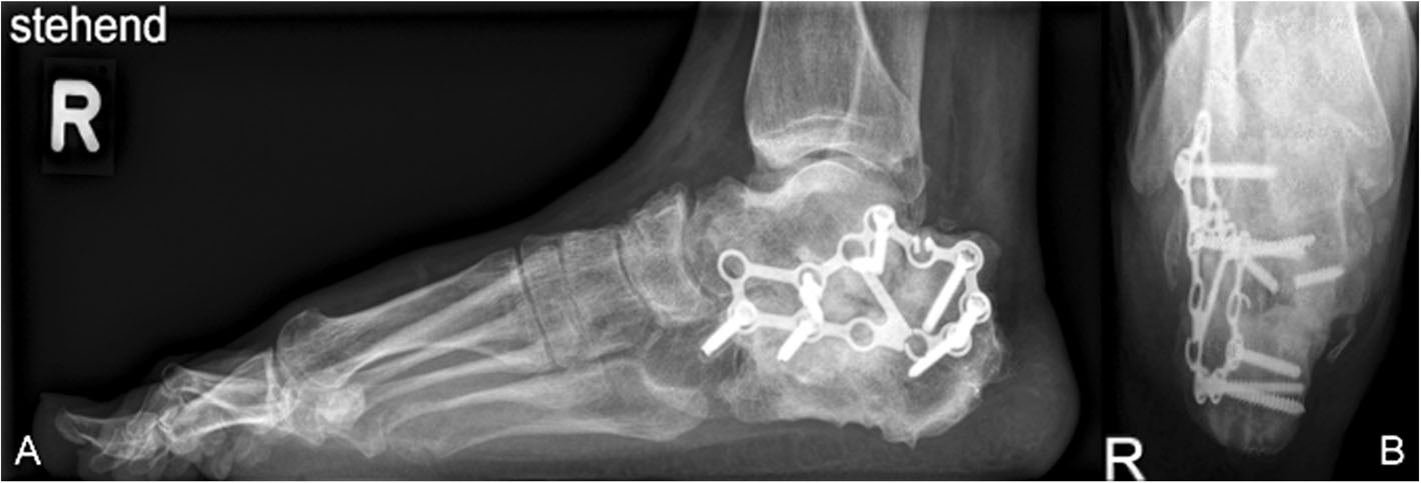
Case 2 Radiographs at follow-up Weightbearing right foot lateral (A) and calcaneus axial (B) 27 months post-surgery, AOFAS: 53

Five fractures (all Sanders IV and closed) were treated with primary subtalar fusion. The decision was made by the attending surgeon according to fracture severity and patient profile. Four of the five patients had concomitant injuries in the involved foot or lower leg. Primary fusion was associated with a high revision rate of 60% (3/5). Two patients needed subsequent revision-arthrodesis due to non-union (both smokers) and one patient a wound revision and partial implant removal of one of two subtalar arthrodesis screws due to deep infection (non-smoker). One arthrodesis (in a smoker) had to be revised twice due to non-union. Only one primary fusion healed without adverse events within 4 months (in a healthy non-smoker). The fifth patient (smoker) developed chronic pain without signs of non-union in the CT scan 6 months after the primary arthrodesis. Figures 6, 7, 8, 9 and 10 show one of the cases that was treated with a primary subtalar arthrodesis. Table 4 shows the mode of fixation and outcome in correlation to fracture severity.

**Table 4:** Mode of fixation and outcome in correlation to fracture severity

Sanders	Method of fixation	Number of feet	AOFAS mean if *n* > 1 (range)	Secondary arthrodesis (n)
I (*n* = 3)	ORIF plate	1	97	0
	ORIF screws	2	100	0
II (*n* = 18)	ORIF plate	15	76.8 (52–100)	6
	ORIF screws	1	72	0
	K-wires	1	72	0
	ExFix	1	50	0
III (*n* = 32)	ORIF plate	27	75.5 (46–97)	3
	ORIF screws	2	69.0 (40–98)	0
	K-wires	2	84.5 (84–85)	0
	ExFix	1	98	0
IV (*n* = 52)	ORIF plate	38	74.9 (52–97)	8
	ExFix	9	60.0 (33–70)	1
	Primary fusion	5	49.7 (30–66)	na

na: not applicable.

The mean preoperative Boehler angle was one degree (range − 70°- 45°) and improved by a mean of 23 degrees (range − 17-95°). There was a clear difference in mean preoperative Boehler angle depending on the Sanders type: Sanders II: 11.6° (range − 11° - 44° max), Sanders III: 1.9° (range - 44°- 30°), Sanders IV: −5° (range - 70 - 45°).

The postoperative Boehler angle improved significantly in all subgroups (*p* < 0.01). The subgroup of Sanders IV fractures showed a tendency of a decrease in the Boehler angle between the postoperative and the follow-up X-rays without statistical significance (*p* = 0.09). Patients with a decrease in Boehler angle had significantly lower AOFAS hindfoot scores (*p* < 0.01).

Complications occurred in 37 out of 129 ft (29%). Postoperative complications included infection, disturbed wound healing, hardware failure, and non-union. At 11%, disturbed wound healing was the most commonly reported complication (14/129) followed by deep infection 5% (6/129). Disturbed wound healing was defined as wound dehiscence or secretion (defined as persistent production of fluid from the wound) 21 days postoperatively or wound necrosis [20]. Seven patients needed secondary surgery for infections and/or disturbed wound healing. Five of those patients required a plastic-surgical procedure to cover the soft tissue defect. Seven of the fifteen patients (7/15, 47%) with infection/disturbed wound healing had Sanders IV fractures. Non-union 4% (5/129) occurred in three patients after ORIF and in two after primary fusion. All non-unions occurred in smokers (*p* = 0.02).

The mean time to return to work was nine months (range 1–100). Twenty-seven patients were not able to return to their former occupation. Table 5 shows the time to return to work depending on the pre-accident degree of manual labour.

**Table 5:** Return to work depending on pre-injury degree of manual labor and Sanders classification.

Degree of physical work	Sanders	n	return to old job	mean time (months)	return to any job	mean time (months)
Light	I	0	0	0	0	0
	II	1	1	1	1	1
	III	11	9	4 (1–14)	9	4 (1–14)
	IV	8	8	6 (3–12)	8	6 (3–12)
	unknown	4	4	3 (2–4)	4	3 (2–4)
	total	24	22	4	22	4
Moderate	I	2	2	8 (4–12)	2	8 (4–12)
	II	8	5	8 (2–18)	8	7 (2–18)
	III	8	5	6 (2–18)	7	5 (2–18)
	IV	15	8	14 (3–48)	10	13 (3–48)
	unknown	13	9	7 (1–18)	9	7 (1–18)
	total	46	29	9	36	8
Heavy	I	1	1	4	1	4
	II	5	3	7 (3–14)	4	7 (3–14)
	III	7	4	30 (5–100)	6	21 (3–100)
	IV	7	3	20 (4–51)	5	21 (4–51)
	unknown	4	1	7	2	16
	total	24	12	17	18	16

The mean time to return to work was highest for patients with Sanders III (30 months, range 5–100 months) and IV fractures (20 months, range 4–51 months), who worked in physically demanding professions. The numbers in each subgroup were too small to reach statistical significance.

Seventy-six percent (87/114) of our patients required subsequent surgical interventions. Implant removal, performed on 86 ft (67%), made up the majority of those. Twenty-two subtalar arthrodeses (18%) were performed 6–80 months (mean 29 months) after the primary operation. Subtalar arthrodesis rates in correlation to fracture severity are shown in Table 6.

**Table 6:** Rate of subtalar arthrodesis

Sanders	Rate of subtalar arthrodesis n
I (n = 3)	0 (0%)
II (n = 18)	6 (33%)
III (n = 32)	3 (9%)
IV (n = 52)*	14 (27%)
unknown (*n* = 24)	4 (17%)

* Including 5 primary subtalar arthrodesis

Three secondary arthrodesis (14%, all smokers) had to be revised due to symptomatic non-union after a mean of 9 months (range 6.5–12 months). Logistic regression did not show independent risk factors for subsequent arthrodesis.

Of the feet that had not undergone subtalar arthrodesis at the time of follow up and were followed up clinically and radiologically, 52 (57%) showed clinical and radiological signs (sclerosis, joint line narrowing, subchondral cysts, and osteophytes) of subtalar osteoarthritis. Criteria for having clinical signs was to show two or more typical symptoms (weather dependent worsening of symptoms, start-up pain in the morning or after rest, pain at night) or one in addition to a severe reduction of subtalar ROM and/or swelling and pain with activity. Ten patients had radiologic signs without being symptomatic. Of the 33 patients interviewed via telephone ten reported no clinical signs of osteoarthritis, nine had one and 12 reported two or more symptoms of subtalar osteoarthritis. Two patients did not answer the question.

### Functional outcome scores

The functional outcome measures were taken postoperatively at the time of the latest follow-up (Table 7).

**Table 7:** AOFAS hindfoot scores

AOFAS	Mean (range)
Total (*n* = 90)	73.5 (30–100)
Sanders I (n = 2)	98.5 (97–100)
Sanders II (*n* = 13)	74.0 (50–100)
Sanders III (n = 26)	76.6 (40–98)
Sanders IV (n = 32)	70.2 (30–97)
unknown (*n* = 17)	71.9 (37–100)

In the three primarily fused patients that were examined, the mean AOFAS score was 50 (range 30–66). The six Sanders IV fractures with a completed score that were subsequently fused had a slightly higher mean AOFAS score of 64 (range 33–82). The numbers were too small to reach statistical significance.

The postoperative Boehler angle was a strong independent predictor for the AOFAS hindfoot scores at the time of follow-up (*p* < 0.01). There was a tendency to lower AOFAS scores with increasing age (*p* > 0.05).

### Clinical examination

Clinical follow-up was obtained in 80 patients with 90 fractures. Restricted hindfoot motion was a common finding. Table 8 shows the subtalar range of motion dependent on Sanders classification. Only subtalar joints that had not undergone arthrodesis previous to the examination were considered (*n* = 74).

**Table 8:** Subtalar range of motion depending on Sanders classification

Sanders	Subtalar range of motion		
	none	< 15°	> 15°
I (n = 2)	0	0	2
II (*n* = 10)	3	4	3
III (n = 24)	9	8	7
IV (*n* = 23)	7	14	2
uc (*n* = 15)	4	10	1
total (n = 74)	23	36	15

uc = unclassified.

Hindfoot alignment was clinically assessed in the standing patient. A neutral hindfoot up to 10° valgus was considered normal. Most feet (87%, 77/89) were within the normal range. Five feet had valgus malalignment (15–30°), seven varus (5–10°). We could not assess hindfoot alignment in one patient as he was not able to fully weight bear at the time of examination due to an unrelated injury of the contralateral ankle.

Heel width difference was assessed by letting the patient stand on a piece of paper and tracing the outline of both heels with a pen. The widest part was measured and compared to the contralateral side. The measurement was done in all but two patients. We only considered patients with unilateral injuries that had an uninjured side to compare to. Thirty out of 68 patients (44%) showed a heel width difference between 0.5-1 cm; three patients (4%) had a difference > 1 cm.

All feet were examined for tenderness to palpation. 58 of 90 (64%) felt no pain. Lateral pain was most common among our patient collective (*n* = 26, 29%).

The question if their shoe size has changed after the injury was answered by 104 out of the 114 patients. Thirty-one patients (30%) answered the question with yes (0 Sanders I, 5 Sanders II, 7 Sanders III, and 16 Sanders IV), seventy-three with no.

Orthopaedic insoles were used by 21% (23 out of 112) of all patients. Seventeen percent of all patients stated the need for orthopaedic shoes (19 out of 112). Two patients did not answer the question.

### SF-36

**Fig. 11:**
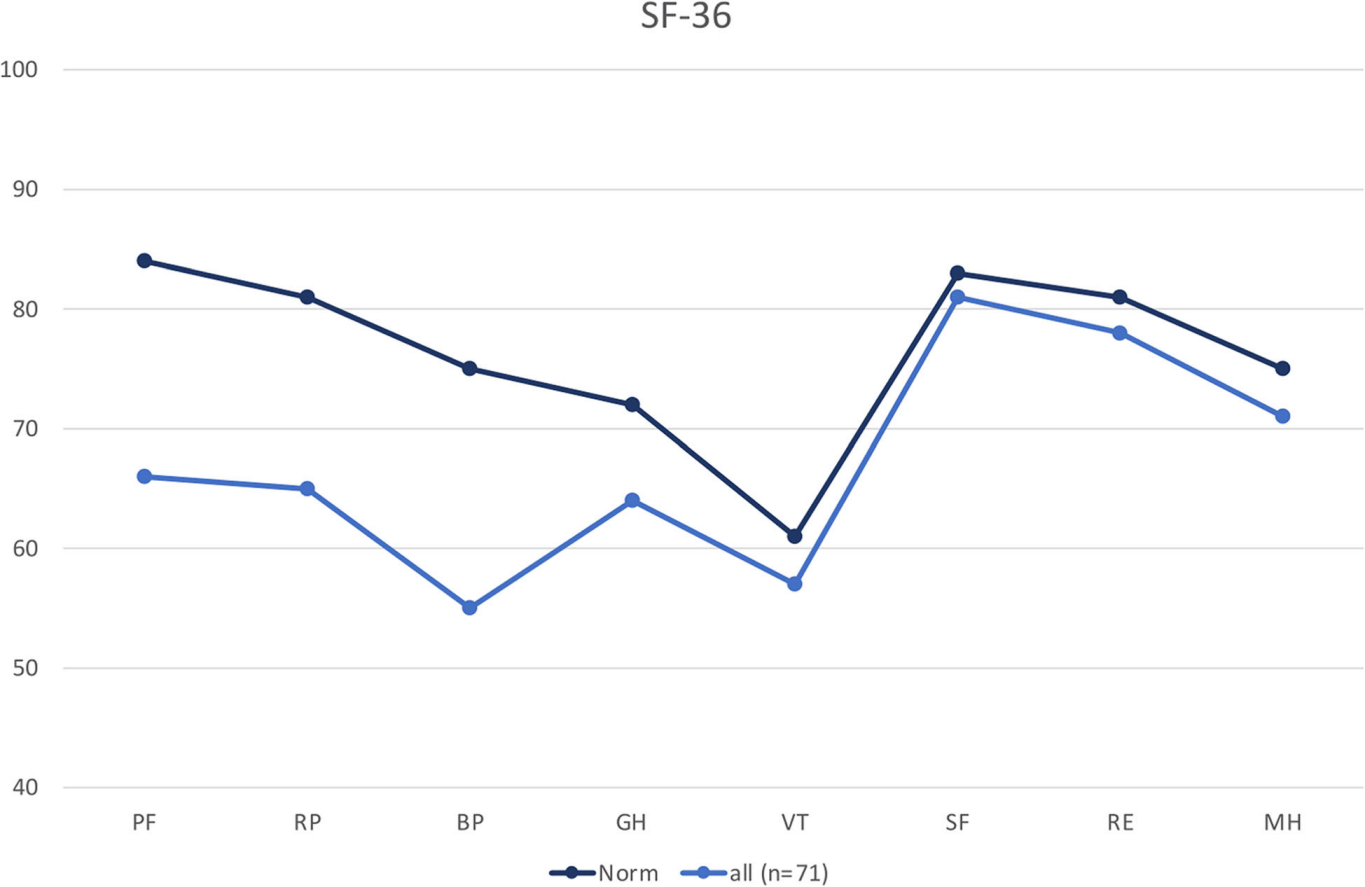
SF-36 normative data compared to all patients with surgically treated calcaneal fractures. PF: Physical functioning. RP: Role physical. BP: Bodily pain. GH: General health. VT: Vitality. SF: Social functioning. RE: Role Emotional. MH: Mental health

**Fig. 12:**
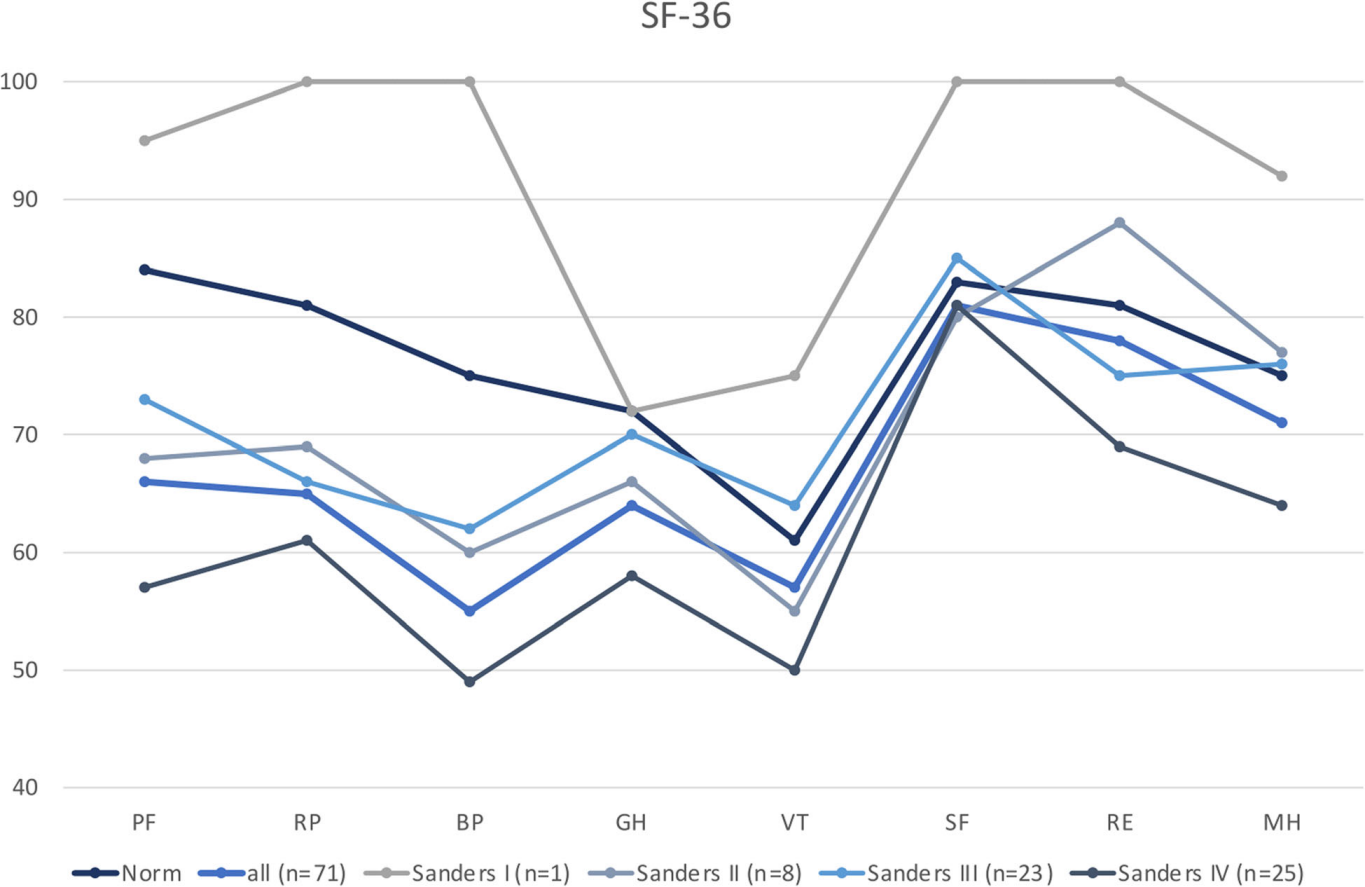
SF-36 normative data compared to patients with surgically treated calcaneal fractures depending on Sanders classification. PF: Physical functioning. RP: Role physical. BP: Bodily pain. GH: General health. VT: Vitality. SF: Social functioning. RE: Role emotional. MH: Mental health

The SF-36 questionnaire was completed by 71 patients. Overall the two domains with scores furthest from the normative data were physical functioning and bodily pain. Social functioning is the domain least influenced by surgically treated calcaneal fractures. In all domains patients with Sanders IV fractures reached the lowest scores, except for social functioning where patients with Sanders II fractures scored slightly worse, but still close to the norm. Figures 11 and 12 show the scores of the eight SF-36 domains in all calcaneal fractures and scores dependent on Sanders classification in comparison with the normative data.

In addition to the eight scale scores the physical component summary (PCS) scores and mental component summary (MCS) scores were calculated and compared [21]. Patients who were treated for a calcaneus fracture classified Sanders II-IV showed below-average PCS scores. Only patients with Sanders IV fractures showed below-average MCS scores (Table 9).

**Table 9:** SF- 36 Physical component summary (PCS) score and mental component summary (MCS) score.

Sanders	PCS	MCS
I (*n* = 1)	55.7	57.7
II (n = 10)	43.3	51.2
III (*n* = 22)	45.6	52.3
IV (n = 23)	40.9	48.1
uc (*n* = 14)	40.5	51.9

Average norm: 50, uc: unclassified.

## Discussion

Intra-articular calcaneal fractures are serious injuries that are associated with prolonged functional limitations in many patients.

The occurrence of posttraumatic subtalar arthritis varies greatly in literature from 2.5% in Poeze’s systematic review to 100% in the long term (10y) follow up of Makki et al. [22, 23]. We assume the great range is partly due to dissimilar follow-up times as well as the variety of criteria applied by the different authors (radiological signs, symptoms, or need for fusion). Seventy percent of our patients had clinical and radiological signs of subtalar arthritis. The subsequent subtalar fusion rate after ORIF was 18% in our patients which is higher than the previously reported ranges of 0% [2, 24] to 12% [17]. The higher fusion rate in our patients might be due to the high percentage of Sanders IV fractures (40%). We were not able to identify independent risk factors for the need for subtalar fusion after operative treatment of intra-articular calcaneal fractures.

Primary arthrodesis which was performed in five patients with Sanders IV fractures was associated with high revision rates of 60% and non-union rates of 40%. Buckley et al. found no statistically significant difference in his randomized controlled trial comparing 14 patients that received primary subtalar fusions and 17 that received ORIF for Sanders IV fractures [25]. The authors excluded patients that continued to smoke and those with concomitant injuries. Since 3 out of 5 patients in our analysis that were primarily fused were smokers and 4 had additional injuries to the foot or lower leg, patient selection might be the cause for different outcomes. Potenza et al. published a mean AOFAS of 70, 12 months after primary subtalar fusion, and of 85 after 53 months in 6 patients (7 ft) with Sanders IV fractures. The average time to fusion was 3 months and return to work 100% (no patient doing heavy work) [26]. Compared to the patients who received subtalar fusion after ORIF of a Sanders IV fracture in our collective, mean AOFAS (64 vs. 50) was lower after primary fusion. There was no statistical significance though, because of the small number of patients.

Complication rates are known to be high ranging from 17.3 to 29% in the literature [24, 27]. Recent studies show lower complication rates with minimally invasive techniques compared to open reduction and internal fixation over an extensile lateral approach (4.8% vs. 20.8%) [28]. In our study, we found an overall complication rate of 29%. Disturbed wound healing (11%) and infection (5%) occurred most commonly. Non-union (4%) developed significantly more often in smokers. Part of our high complication rate might be explained through the fact that we had a high percentage of Sanders IV fractures (40%) and smokers (41%) in our collective.

The Boehler angle as an outcome predictor has been described in numerous studies [23, 27, 29–31]. Rammelt et al. stated that a reduction of the Boehler angle to less than 30% of the healthy side is associated with worse outcomes [27]. Basile et al. found a positive correlation between the restoration of the Boehler angle and postoperative AOFAS and VAS scores in elderly patients [31]. Su et al. investigated the role of the Boehler angle in assessing fracture severity and functional outcome and found a correlation of fracture severity with the preoperative Boehler angle and a correlation of the postoperative Boehler angle with the AOFAS score [30]. We also noticed that the postoperative Boehler angle is a strong independent predictor for the AOFAS hindfoot score at the time of follow up. Additionally, we could show that a decrease in the Boehler angle between the postoperative and follow-up X-rays, indicating a loss of reduction, is associated with a significantly worse outcome.

Limitations of our study are the retrospective study design, different methods of treatment, and the small population number in certain subgroups. The use of the AOFAS score as a primary outcome measure is another limitation, because although it is widely used the AOFAS score has not been adequately validated. The relatively large number of surgeons performing a technically demanding procedure (1.2 fractures/surgeon/year) could be another limitation as previous studies have shown a correlation between institutional fracture load and outcome [22].

Residual step-offs in the articular surface where not included in the analysis of prognostic factors as we did not conduct postoperative CT scans or intraoperative subtalar arthroscopy to accurately measure that factor. The restoration of Boehler angle is only an indirect measure of the quality of joint reduction. Moreover, the Boehler angle has a great individual variability of 20–40°. Without measuring the unfractured side’s Boehler angle no statement can be made as to whether we managed to restore the patient’s individual Boehler angle.

Strengths are the long follow-up of ø 91 months with a relatively high overall patient number for a monocentric study. We were able to conduct follow-up on 114 out of 140 patients (81%) which is superior to previously published long term follow-up studies.

## Conclusions

Due to the unfavorable outcome in our patient collective and to the fact that we could not make out clear risk factors for subsequent fusion, we indicate primary subtalar fusion with great caution. We believe the restoration of the anatomy is paramount in the treatment of displaced intraarticular calcaneus fractures in all patients without contraindications for surgical treatment. This and other studies led to changes in our surgical approach. At our institution most fractures are currently treated with less- and minimally- invasive techniques. The lateral extensile approach is avoided whenever possible. We routinely use intraoperative 3D-imaging to ensure adequate reduction is achieved. Smoking status should be considered and cessation strongly recommended.

## Data Availability

The datasets used and analysed during the current study are available from the corresponding author on reasonable request.

## Abbreviations

AOFAS hindfoot score: American Orthopaedic Foot and Ankle Society hindfoot score
SF-36: Short Form 36 Health Status Survey
ROM: range of motion
py: pack-years
ORIF: open reduction and internal plate fixation
PCS score: physical component summary scores
MCS score: mental component summary scores
MVA: motor vehicle accident

## References

[1] Eastwood DM, Phipp L (1997). Intra-articular fractures of the calcaneum: why such controversy?. Injury..

[2] Griffin D, Parsons N, Shaw E, Kulikov Y, Hutchinson C, Thorogood M, et al. Operative versus non-operative treatment for closed, displaced, intra-articular fractures of the calcaneus: randomised controlled trial. BMJ [Internet]. 2014 Jan [cited 2015 Jan 17];349:g4483. Available from: http://www.pubmedcentral.nih.gov/articlerender.fcgi?artid=4109620&tool=pmcentrez&rendertype=abstract 10.1136/bmj.g4483 PMC4109620 25059747

[3] Hildebrand KA, Buckley RE, Mohtadi NG, Faris P (1996). Functional outcome measures after displaced intra-articular calcaneal fractures. J Bone Joint Surg Br.

[4] Mitchell MJ, McKinley JC, Robinson CM (2009). The epidemiology of calcaneal fractures. Foot..

[5] Sanders R (2000). Displaced intra-articular fractures of the calcaneus. J Bone Joint Surg Am.

[6] van Tetering E. A A, Buckley RE. Functional outcome (SF-36) of patients with displaced calcaneal fractures compared to SF-36 normative data. Foot ankle Int / Am Orthop Foot Ankle Soc [and] Swiss Foot Ankle Soc. 2004;25(10):733–8. 10.1177/107110070402501007 15566705

[7] Dhillon MS, Prabhakar S (2017). Treatment of displaced intra-articular calcaneus fractures: a current concepts review. SICOT-J..

[8] Parmar H V, Triffitt PD, Gregg PJ. INTRA-ARTICULAR FRACTURES OF THE CALCANEUM TREATED OPERATIVELY OR CONSERVATIVELY A PROSPECTIVE STUDY. THE JOURNAL OF BONE AND JOINT SURGERY. 1993;75(6):932-7 10.1302/0301-620X.75B6.8245085 8245085

[9] Ibrahim T, Rowsell M, Rennie W, Brown AR, Taylor GJS, Gregg PJ (2007). Displaced intra-articular calcaneal fractures: 15-year follow-up of a randomised controlled trial of conservative versus operative treatment. Injury..

[10] Sharma V, Dogra A (2011). Sanders type II calcaneum fractures-surgical or conservative treatment? A prospective randomized trial. J Clin Orthop Trauma.

[11] Thordarson DB, Krieger LE (1996). Operative vs. nonoperative treatment of intra-articular fractures of the calcaneus: a prospective randomized trial. Foot Ankle Int.

[12] E.C. Rodriguez-Merchan E. Galindo. Intra-articular displaced fractures of the calcaneus Operative vs non-operative treatment. Int Orthop. 1999;23(1):63-5. doi: 10.1007/s002640050307. 10.1007/s002640050307 PMC3619771 10192023

[13] Howard JL, Buckley R, McCormack R, Pate G, Leighton R, Petrie D (2003). Complications following Management of Displaced Intra-Articular Calcaneal Fractures: a prospective randomized trial comparing open reduction internal fixation with nonoperative management. J Orthop Trauma [Internet].

[14] Bahari Kashani M, Reza Kachooei A, Ebrahimi H, Taghi Peivandi M, Amelfarzad S, Bekhradianpoor N, et al. Comparative study of peroneal tenosynovitis as the complication of intraarticular calcaneal fracture in surgically and non-surgically treated patients. Iran Red Crescent Med J. 2013;15(10). 10.5812/ircmj.11378 PMC3950774 24693362

[15] Buckley R, Tough S, McCormack R, Pate G, Leighton R, Petrie D (2002). Operative compared with nonoperative treatment of displaced intra-articular calcaneal fractures: a prospective, randomized, controlled multicenter trial. J Bone Joint Surg Am.

[16] Author C, Hadi Nouraei M, Mostafa Moosa F. A r c h i v e o f S I D Operative compared to non-operative treatment of displaced intra-articular calcaneal fractures * [Internet]. Vol. 16, J Res Med Sci. 2011. Available from: www.SID.ir PMC3263077 22279476

[17] Agren P-H, Wretenberg P, Sayed-Noor AS (2013). Operative versus nonoperative treatment of displaced intra-articular calcaneal fractures: a prospective, randomized, controlled multicenter trial. J Bone Joint Surg Am.

[18] Sanders R, Vaupel ZM, Erdogan M, Downes K (2014). Operative treatment of displaced intraarticular calcaneal fractures: long-term (10-20 years) results in 108 fractures using a prognostic CT classification. J Orthop Trauma.

[19] Rammelt S, Zwipp H, Schneiders W, Dürr C. Severity of injury predicts subsequent function in surgically treated displaced intraarticular calcaneal fractures. In: Clinical Orthopaedics and Related Research. Springer New York LLC; 2013. p. 2885–98. 10.1007/s11999-013-3062-z PMC3734437 23690151

[20] Müller AM, Toepfer A, Harrasser N, Haller B, Walther M, von Eisenhart-Rothe R, et al. Significant prevalence of peripheral artery disease in patients with disturbed wound healing following elective foot and ankle surgery: Results from the ABI-PRIORY (ABI as a PRedictor of Impaired wound healing after ORthopedic surgerY) trial. Vasc Med (United Kingdom). 2019; 10.1177/1358863X19883945 32366205

[21] Laucis NC, Hays RD, Bhattacharyya T (2014). Scoring the SF-36 in orthopaedics: a brief guide. J Bone Jt Surg - Am Vol.

[22] Poeze M, Verbruggen JPAM, Brink PRG (2008). The relationship between the outcome of operatively treated calcaneal fractures and institutional fracture load. J Bone Jt Surgery-American Vol [Internet].

[23] Makki D, Alnajjar HM, Walkay S, Ramkumar U, Watson AJ, Allen PW (2010). Osteosynthesis of displaced intra-articular fractures of the calcaneum: a long-term review of 47 cases. J Bone Joint Surg Br..

[24] De Boer AS, Van Lieshout EMM, Den Hartog D, Weerts B, Verhofstad MHJ, Schepers T. Functional outcome and patient satisfaction after displaced intra-articular calcaneal fractures: a comparison among open, percutaneous, and nonoperative treatment. J Foot Ankle Surg. 2014. 10.1053/j.jfas.2014.04.014 24891090

[25] Buckley R, Leighton R, Sanders D, Poon J, Coles CP, Stephen D, et al. Open reduction and internal fixation compared with ORIF and primary Subtalar arthrodesis for treatment of Sanders type IV calcaneal fractures: a randomized multicenter trial. J Orthop Trauma. 2014;28. 10.1097/BOT.0000000000000191 24983433

[26] Potenza V, Caterini R, Farsetti P, Bisicchia S, Ippolito E (2010). Primary subtalar arthrodesis for the treatment of comminuted intra-articular calcaneal fractures. Injury..

[27] Rammelt S, Zwipp H (2004). Calcaneus fractures: Facts, controversies and recent developments. Injury..

[28] Peng Y, Liu J, Zhang G, Ji X, Zhang W, Zhang L (2019). Reduction and functional outcome of open reduction plate fixation versus minimally invasive reduction with percutaneous screw fixation for displaced calcaneus fracture: a retrospective study. J Orthop Surg Res.

[29] Eckstein C, Kottmann T, Füchtmeier B, Müller F (2016). Long-term results of surgically treated calcaneal fractures: an analysis with a minimum follow-up period of twenty years. Int Orthop.

[30] Su Y, Chen W, Zhang T, Wu X, Wu Z, Zhang Y. Bohler’s angle’s role in assessing the injury severity and functional outcome of internal fixation for displaced intra-articular calcaneal fractures: A retrospective study. BMC Surg. 2013;13(1). 10.1186/1471-2482-13-40 PMC3849198 24330592

[31] Basile A (2010). Operative versus nonoperative treatment of displaced intra-articular calcaneal fractures in elderly patients. J Foot Ankle Surg.

